# NaviCom: a web application to create interactive molecular network portraits using multi-level omics data

**DOI:** 10.1093/database/bax026

**Published:** 2017-04-02

**Authors:** Mathurin Dorel, Eric Viara, Emmanuel Barillot, Andrei Zinovyev, Inna Kuperstein

**Affiliations:** 1 Institut Curie, 26 rue d'Ulm, F-75005 Paris, France; 2 Inserm, U900 F-75005, Paris France; 3 Mines Paris Tech, F-77305 Cedex Fontainebleau, France; 4 PSL Research University, Paris F-75005, France; 5 Ecole Normale Supérieure, 46 rue d’Ulm, Paris, France; 6 Institute of Pathology and Institute for Theoretical Biology, Charite - Universitätsmedizin Berlin, Chariteplatz 1, Berlin 10117, Germany; 7 France Sysra F-91330, Paris, France

## Abstract

Human diseases such as cancer are routinely characterized by high-throughput molecular technologies, and multi-level omics data are accumulated in public databases at increasing rate. Retrieval and visualization of these data in the context of molecular network maps can provide insights into the pattern of regulation of molecular functions reflected by an omics profile. In order to make this task easy, we developed NaviCom, a Python package and web platform for visualization of multi-level omics data on top of biological network maps. NaviCom is bridging the gap between cBioPortal, the most used resource of large-scale cancer omics data and NaviCell, a data visualization web service that contains several molecular network map collections. NaviCom proposes several standardized modes of data display on top of molecular network maps, allowing addressing specific biological questions. We illustrate how users can easily create interactive network-based cancer molecular portraits via NaviCom web interface using the maps of Atlas of Cancer Signalling Network (ACSN) and other maps. Analysis of these molecular portraits can help in formulating a scientific hypothesis on the molecular mechanisms deregulated in the studied disease.

**Database URL: **NaviCom is available at https://navicom.curie.fr

## Introduction

Today's biology is largely data-driven, thanks to high-throughput technologies that allow investigating molecular and cellular aspects of life at large scale. These technologies comprise microarray platforms, next-generation sequencers, mass spectrometers, or interaction screens, producing multi-level omics data such as gene and protein expression, mutational profiles, epigenetic landscapes, etc (1, 2). Samples of diseased tissues, especially in cancer, are routinely characterized by these high-throughput techniques, and multi-level omics data are accumulated in public and proprietary databases. >20 000 tumor samples have been profiled so far by two main international efforts, The Cancer Genome Atlas (TCGA, www.cancergenome.nih.gov) and the International Cancer Genome Consortium (ICGC, www.icgc.org). Multiple cancer omics datasets are available through the Gene Expression Omnibus (GEO, www.ncbi.nlm.nih.gov/geo) and the cBioPortal web interface (www.cbioportal.org) (3). An integrated analysis of different data types can provide the most comprehensive picture on the status of the studied sample: however, this represents a challenge because of the complex cross-correlations in the structure of multi-level omics data (4). Data visualization is a possible solution to obtain an integrated overview of the data and understanding their characteristic patterns. Making biological sense out of the molecular data requires visualizing them in the context of cell signalling processes (5, 6). A lot of information about molecular mechanisms is available in the scientific literature, and is also integrated into signalling pathway databases. Those signalling pathway databases, such as Reactome (7), KEGG PATHWAYS (8), Spike (9), PathwaysCommons (10), ConsensusPathDB (11), SIGNOR (12), Atlas of Cancer Signalling Network (ACSN) (13) etc, vary in the approach to depict molecular interactions and in the level of details of biological processes representation (14). Several data visualization tools address the problem of exploiting omics data in the context of biological pathways and networks. Such tools as ipath (15), BioCyc (16), Pathway projector (17), NetGestalt (18), several plugins in Cytoscape (19, 20) or NaviCell (21) can be used together with a number of pathway databases. In addition, some pathway databases contain integrated data visualization tools such as the Reactome Analysis tools or FuncTree for KEGG PATHWAYS (22).

Nevertheless, there remain several poorly addressed problems in the field. First, exploiting network maps and pathway databases for the visualization of multi-level omics data faces the challenge of compatibility between the databases, data types and visualization methods (5, 14). Users without computational biology background may face difficulties when choosing the most appropriate tool for their purposes (4). The second bottleneck for an unexperienced user lies in accessing public omics data resources and retrieving the data, for instance in order to compare the original results obtained by a research group with publicly available molecular profiles (2). There exists a number of data visualization tools exploiting different approaches for data integration that we discuss and compare below. However, current tools rarely support automated and user-friendly import of large datasets from omics data resources and displaying them on top of molecular network maps. The third challenge is providing to the user the most informative and readable combination of data visualization settings (23).

To fill these gaps, we developed NaviCom, a tool for automatically fetching and displaying several omics data types on top of the large network maps, using optimized pre-defined data visualization modes. NaviCom connects cBioPortal to NaviCell and allows visualizing various high-throughput data types simultaneously on a network map. To highlight the possibilities provided by this tool, we demonstrate how multi-level cancer omics data from cBioPortal (3) are automatically visualized on the molecular network maps available in ACSN (13) and NaviCell (24) collections.

Integrating together different type of data allows to create the comprehensive molecular portraits of cancer, identifying specific patterns in the data that may lead to better disease subtypes classification (24, 25, 26). Visualization of the high-throughput data in the context of biological networks is an essential step helping to grasp the general trends of data distribution across cell molecular mechanisms represented as signalling network maps (27). Visualizing data at different zoom levels of cell signalling can be also helpful for assessment of patterns, structures and functional network modules deregulated in the disease, that can guide in narrowing down the areas of interest for further detailed study of the molecular mechanisms.

NaviCom allows the user to create complex interactive molecular portraits of cancers based on simultaneous integration and analysis of multi-level omics data in the context of comprehensive signalling network maps. This broaden the possibilities of data interpretation, allowing not only to pattern the traits of changes in the data, but also to understand involvement of specific molecular mechanisms associated with the studied disease.

## Materials and methods

### Data source and type

We use available omics data from cBioPortal (3) (www.cbioportal.org), a web resource for the exploration of cancer genomic datasets from TCGA project and several other projects. The studies in the cBioPortal database contain large-scale cancer data sets including expression data for mRNA, microRNA, proteins; mutation, gene copy number, methylation profiles and beyond. cBioPortal data can be extracted per gene or per patient using the R package cgdsr, an R connection to the Cancer Genomic Data Server API, a REST-based programming interface.

Datasets used for application examples described below are: Breast Invasive Carcinoma (TCGA, Nature 2012), Acute Myeloid Leukemia (AML) (TCGA, NEJM 2013), Adenocortical Carcinoma (TCGA Provisional), Ovarian Serous Cystadenocarcinoma (TCGA, Nature 2011), Glioblastoma (TCGA, Cell 2013), Sarcoma (TCGA, Provisional)

### Signalling network maps

Any map prepared in NaviCell format can be used for visualization of data via NaviCom. To demonstrate this feature, we provide three visualization examples on maps with different characteristics: i). The Atlas of Cancer Signalling Network (ACSN, https://acsn.curie.fr) (13) that contains a comprehensive description of cancer-related mechanisms retrieved from the recent literature, following the hallmarks of cancer (28). Construction and update of Atlas of Cancer of Signalling Network (ACSN) is done using CellDesigner tool (29), involving manual mining of molecular biology literature. ii). Alzheimer’s map (30) created using Cell Designer tool (29). iii). Ewing’s Sarcoma signalling map (31) created in map drawing tool of Cytoscape. Last two maps are part of the NaviCell collection (https://navicell.curie.fr/pages/maps.html) (24). The majority of the maps available in ACSN and NaviCell collection are composed of multiple sub-maps (functional modules) that represent individual signalling pathways or well-defined molecular processes (13, 24). This hierarchical structure provides a possibility to visualize data at different depth of signalling representation, starting from general overview of data on the complete maps up to the data visualization at the level of individual processes or pathways.

### NaviCom software design

**Figure 1.**
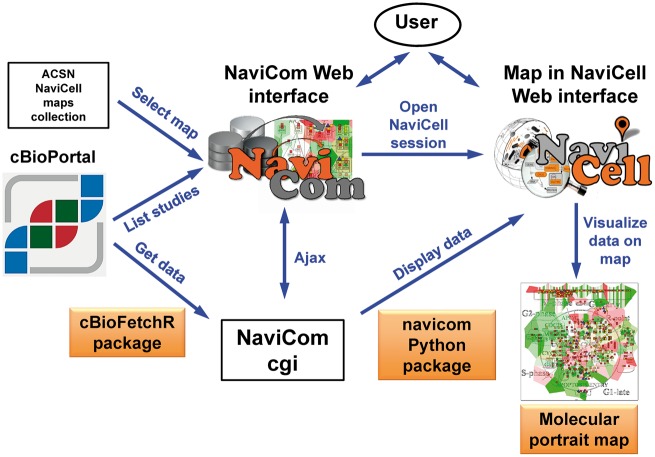
General architecture of NaviCom. The NaviCom interface provides the user with an updated list of studies from cBioPortal and links to ACSN and NaviCell maps collections. When visualization is requested, NaviCom starts a new NaviCell session and calls a cgi on the server. The cgi downloads cBioPortal data to the NaviCell session and displays them to generate the molecular portrait selected by the user.

NaviCom is a web interface to the python package navicom and the R package cBioFetchR to import, format and display data from cBioPortal on the signalling maps using NaviCell visualization procedures (Figure 1). The interaction with cBioPortal is performed using our R package cBioFetchR, an easy to use wrapper around the R package cgdsr. The interaction with NaviCell is managed by our python module navicom, which defines optimized visualisation modes for various types of high-throughput data. The list of studies available on cBioPortal is generated dynamically when the NaviCom web page is opened, thus presenting up-to-date information to the user. NaviCom also extracts the number of samples, and the types of data available in each dataset. In addition, NaviCom allows selecting individual samples from datasets and to visualize data using a single sample of interest on the maps. Once the visualisation is requested, NaviCom accesses a cached copy of the dataset, or downloads it directly from cBioPortal with cBioFetcher to generate this local copy. The data are then loaded in NaviCell by the navicom module according to the visualization mode that is requested through NaviCom. NaviCom provides a default visualization setting (detailed below) for simultaneous integration of the data into the big comprehensive maps of molecular interactions. Once chosen, these settings are applied automatically, significantly reducing the time required to perform the visualization comparing to manual mode. It also allows launching the visualization of several datasets on different maps in parallel. In addition, since the whole dataset is already imported to NaviCell in a form of data tables, the user also may apply different types of analyses provided by the NaviCell environment. Finally, the tool can be run using the command line that opens further possibilities for data analysis (Supplementary Table S1).

### NaviCom data visualization

Navicom uses combinations of the visualisations channels available in NaviCell. Those multiple channels offer different ways displaying the data on top of the network map, which allow the user to visualize several layers of information simultaneously on one map in a comprehensive manner.

Data representation through multiple channels correspond to various graphics:

**Barplots** are histograms located near each entity, with the height and color of the bar depending on the value of the data for the entity.
**Heatmaps** consist in plotting colored squares near each node, with the color depending on the value of the data for the node.
**Glyphs** consist of geometrical figures with variable shape, color and size depending on the values of the data for the entity.
**Map staining** is an original display mode for coloring background areas around each entity according to the data value associated to this entity.

### Navicom display settings

NaviCom uses combination of the NaviCell graphics described above, to overlay multiple data types. To avoid time-consuming manual manipulation, the visualization settings are standardized as pre-defined data display modes (Table 1). These NaviCom settings are applied automatically, making the visualization process easy for users. NaviCom allows launching visualization of several datasets on different maps or submaps (modules, pathways) in parallel. The visualization is applied to the entire dataset or using a selected sample, depending on the choice of the user. In addition, since the data and the sample annotation table from cBioPortal are already imported into NaviCell by NaviCom the user can visualise and explore the data in depths. For example, via the interactive interface of NaviCell the user can define various sample subgroups using any characteristics from the sample annotation table (e.g. patient age, disease stage, relapses, type of treatment, survival time, etc.) and visualize the data for those subgroups in the same manner as described above.

**Table 1.** Data display settings in NaviCom

Date type	Visualization mode	Data display	Units
mRNA expression	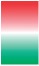	Map staining	Level
Gene copy number	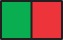	Heat map	Count
Mutation data	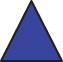	Glyph 1	Frequency
Methylation data	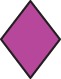	Glyph 2	Intensity
miRNA expression	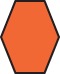	Glyph 3	Level
Protein expression	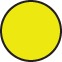	Glyph 4	Level

### There are four major pre-defined NaviCom display modes

Complete data type display mode overlays as many information as possible on the map in order to compare the coherence of the various signals, and to spot the most affected areas on the signalling map. This mode is suitable for exploratory analysis and comparison of samples.

Triple data type display mode (Mutations and genomic data) shows genomic and transcriptomic data together: mRNA expression as map staining, copy number variation as heat map and the mutations as glyph, together showing alterations of each gene at different regulatory levels.

Double data type display mode shows each one of the available data type in the context of mRNA expression as map staining, which allows comparison of the profile in the displayed omics (e.g. methylation) data with the transcriptional status of the gene.

Single data type display mode is the simplest and fastest visualization mode allowing user to focus on distribution of one type of data over the map.

In addition, a user can choose to ‘Export the dataset to NaviCell’, which will grant access to all the datatables of the dataset with a preconfigured display settings on a NaviCell map, to perform different analysis of the data.

### NaviCom access, availability and documentation

The NaviCom web page is freely accessible via http://navicom.curie.fr. cBioFetchR and *navicom* codes are available at https://github.com/sysbio-curie/cBioFetchR and https://github.com/sysbio-curie/navicom respectively. NaviCom, cBioFetchR and *navicom* are distributed under LGPL license. A detailed documentation including introduction into the NaviCom features, code description, manual, tutorial and application examples and suggested scenarios are available at https://navicom.curie.fr/downloads.

## Results

We demonstrate a new tool NaviCom that - optimizes usability and increases compatibility between existing resources. We show how NaviCom solves the problem of connecting the omics data resource cBioPortal and the collection of signalling maps in ACSN and NaviCell; and optimizes data visualization.

We first provide an overview of NaviCom workflow, thus describe the features of NaviCom comparing to other efforts in the field and finalize by NaviCom application examples, including molecular portraits of several cancer types and biological interpretation of the results.

### NaviCom workflow

**Figure 2.**
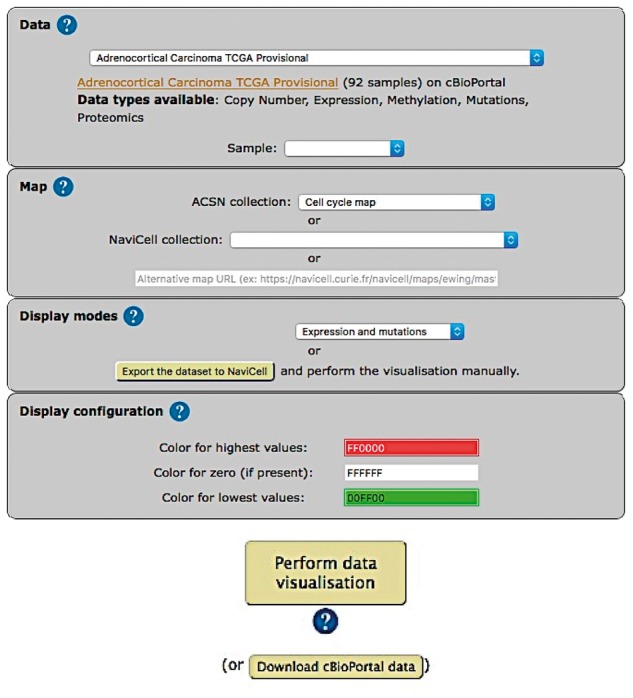
Visualization setting panel of NaviCom.

The web-based user interface for NaviCom makes it easy to display omics data from cBioPortal on interactive maps provided in NaviCell format (http://navicom.curie.fr). The workflow consists of several simple steps (Figure 2): (i**) Data**: selection of the dataset from the list available in cBioPortal. A short summary (types of data and number of samples available) is available for each datasets. Individual samples from the chosen dataset are displayed in the **‘Sample’** drop-down list and can can be selected. (ii) **Map**: selection of the molecular network map or their sub-maps (modules, pathways) from ACSN or NaviCell collections to visualize the data on. The user can import and visualize data via NaviCom on the maps or on the modules, depending on the wish of the user to have a general overview of the data distribution on the map or to go into the depth of the signalling processes at the pathway level displayed on the submaps (modules). Moreover, any type of signalling networks prepared in NaviCell format (23) and displayed in NaviCell collection can be used for data integration and visualization. (iii) Display modes: selection of the data visualization procedure may vary depending on the data available in the data set and the scientific question. (iv) (optional). Display configuration*:* the data display default settings are pre-defined by NaviCom (Table 1): however users may adjust the color gradient settings. Once chosen, these settings are applied on the NaviCell map, significantly reducing the time required to perform the visualization comparing to manual mode. The map with data displayed according to the chosen configurations will be opened in a separate tab containing a NaviCell session.

**Figure 3.**
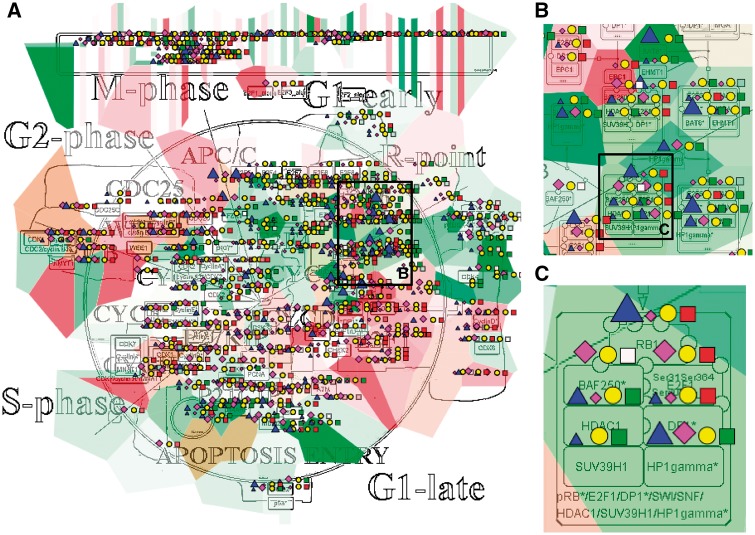
Multi-omics data visualization in Cell Cycle signalling map. Five types of omics data, copy number, expression, methylation, mutations, proteomics, for breast invasive carcinoma dataset from cBioPortal has been displayed on the map using the pre-defined display mode as detailed in Table 1. The values represent average for 825 samples available in the dataset (A) Top level view of data distribution, (B) and (C) Zoom in on individual entities on the map.

The resulting maps with visualized data on top of them are interactive and can be browsed using NaviCell Google Maps-based navigation features (Figure 3). For example, semantic zooming allows the user to interact with the maps starting from the top level view (Figure 3A), where patterns of integrated data can be grasped, up to the most detailed view at the level of individual actors (Figure 3B and C). In addition, visualization of data on module maps zooms in on particularities of molecular regulation at the level of individual molecular processes. This simplifies interpretability of the results and provides additional possibility to retrieve a biological significance from the visualization.

### NaviCom features

NaviCom is characterized by the combination of the following essential and unique features: (i) Accessibility via a user-friendly web interface; (ii) Fully integrated tool allowing automatic fetching and display of various omics data types on molecular network maps at different depth of signalling details representation; (iii) It offers several meaningful scenarios of multi-level omics data visualization. The data pre-defined display modes in NaviCom are optimized for simultaneous visualization of several data types, which makes the visualization process easy for users; iv). The user can import and visualize data via NaviCom on any NaviCell map. NaviCom allows exploiting not only pre-defined but also user-created pathway maps; (v) The tool can be run using the command line, allowing the integration into data analysis pipelines with little efforts; (vi) The resulting ‘colored’ interactive maps with displayed data are characteristic complex molecular portraits of cancer types or molecular portrait of individual patients.

### Comparison of NaviCom to similar tools

We compared NaviCom functionalities to similar tools available in the field (Table 2). For example, Cytoscape (19) is a widely used network visualization software which provides several plugins to analyse data in a network context, and some to perform simple visualization (CyLineUpi, PINA4MSi, CytoHiC), but does not allow overlay of several type of data. The BiNoM (32) plugin currently provides two types of data display: coloring of individual map nodes and map staining using pre-defined ‘territories’ of functional modules for displaying average values of expression for all genes in each module. However, BiNoM requires external CellDesigner screenshots, and does not provide many visualization modes. FuncTree (22) is a web application for analysis and visualization of large scale genomic data. It is linked to KEGG PATHWAYS (8) and offers many functionalities like normalization or enrichment analysis. It can display data as circles of varying diameter, but does not allow the display of several types of data at the same time and requires a lot of interactions from the user. iPath (15) is a web-based tool for the visualization of data on pathways maps where the size and color of the nodes can be set to match various data, which allows a very clear visualization. However, iPath requires the colors and width to be specified as inputs, and provides no processing functionalities. It also lacks a convenient zooming and provides visualization on metabolic maps only. NetGestalt is an advanced tool for integration of multidimentional omics data exploiting simple and easily readable one-dimensional layouts of gene networks (18). It however does not link to any data database, making it necessary to download and pre-process the data to match the input format. Pathway Projector (17) is a web-based pathway browser which allows custom data visualization. It can use KEGG PATHWAYS maps and provides many modes of display for the data. However, this power comes at the cost of a complex interface, which require heavy preprocessing of the data. Reactome (7) is a pathway database which provide mapping of data on its pathway maps. BioCyc (16) is a collection of pathway databases similar to Reactome which provides tools for visualizing multi-omics data on pathway map. However, this visualization is limited to a large scale view of the pathway, which hinders the ability to interpret such visualization for large scale data, and provides only REST API which might be non-trivial to exploit.

**Table 2.** Comparison of NaviCom with similar tools

Feature	Tool							
	REACTOME	Ipath	BioCyc	Pathway projector	FuncTree KEGG PATHWAYS	Cytoscape	NetGestalt	NaviCom
Data fetching			•			•		•
Multi-level data display			•	•		•	•	•
Data display pre-setting	•	•			•		•	•
Interactive maps	•	•				•	•	•
Web interface	•	•	•	•	•		•	•
Reference	7	15	16	17	22	19	18	

The most important advantages of NaviCom web application over existing similar tools is that it combines several essential functions together: allows fetching the data and visualizing several large multi-level omics dataset or selected samples from cBioPortal on top of comprehensive molecular maps and their submaps (modules, pathways) even for inexperienced users. The combination of these features is missing in the currently existing visualization tools. 

### Molecular portraits of cancer

We define a molecular portrait as a visualization of molecular traits of a biological sample or a group of samples on top of a signaling map. We demonstrate different possibilities for data visualization and interpretation using NaviCom depending on the scientific question.

In the first example, we show how visualization on networks helps to grasp the data distribution over the biological processes and to understand the general patterns of processes involvement into a disease. We use the Cell Cycle map from the ACSN collection, created in CellDesigner and presented in the NaviCell format. Figure 3 shows the map with the complete display of the Breast Invasive Carcinoma dataset, consisting of 5 types of omics data. The rich dataset consists of 825 samples, meaning that the aggregated values display provides the most general picture of molecular mechanisms deregulation in the disease, including relatively rare mutations. The data is observed at the top level view of the map, providing the general pattern of data distribution (Figure 3A). The map with displayed data is interactive, data distribution on individual entities can be observed while zooming in (Figure 3B and C).

In the second example, we show data from one patient from the list of Glioblastoma dataset overplayed on the Alzheimer’s disease signalling map from NaviCell collection, that is also created in CellDesigner pathway editor. Three types of data (expression, copy number and mutations) are visualized simultaneously, showing the ‘hot spots’, where the expression variability co-insides with copy number variations and with high frequency of mutations. Thus the module containing MAPK pathway shows that several upstream MAPK pathway members are upregulated at the level of expression and copy number, but also bare mutations in high frequency. Since these are very central kinases in the cell survival mechanisms and have a well known role in tumor survival and proliferation, it will be interesting to investigate the status of their kinase activity. Complementary, the members of ‘Blood-brain barrier (BBB) signalling shows as downregulated and also mutated with different frequencies, together indicating perturbation in the BBB control, the characteristic signature of brain cancers (Supplementary Figure S1).

In addition, any biological network map that can be imported into Cytoscape environment can be also used for data display using NaviCom, as it is shown for the Ewing’s Sarcoma signalling map, demonstrated with the complete display of the Sarcoma dataset (TCGA, Provisional) (Supplementary Figure S1).

**Figure 4.**
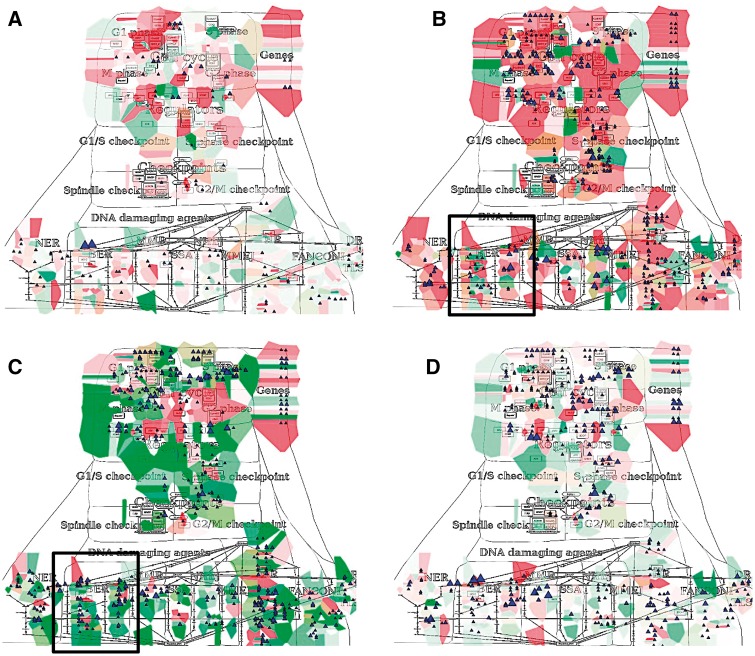
Molecular portraits of cancer types. Expression and mutation data from cBioPortal has been displayed on the DNA repair map using the pre-defined display mode as detailed in Table 1. The values represent average for all samples available in each dataset (A) acute myeloid leukemia, (B) adenocortical carcinoma (C) ovarian serous cystadenocarcinoma and (D) glioblastoma.

**Figure 5.**
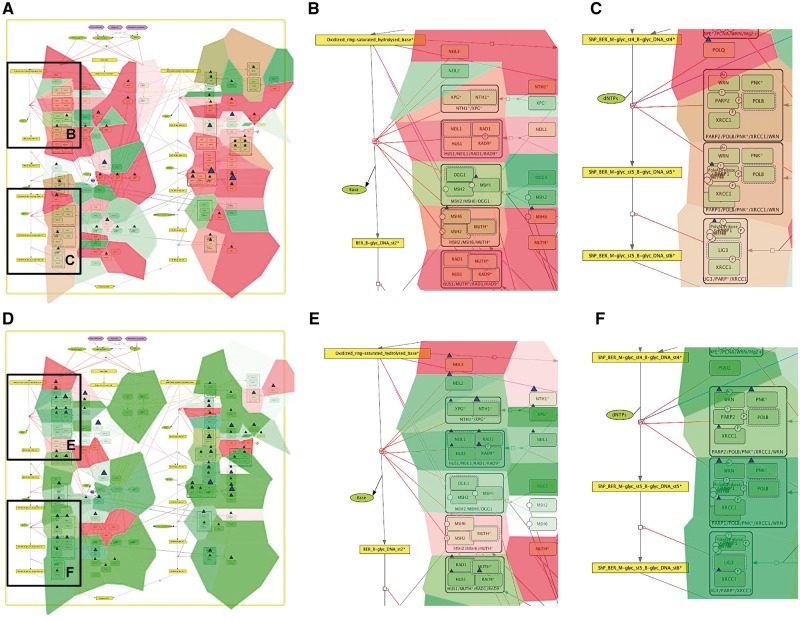
Comparison of base excision repair module regulation in two cancer types. Expression and mutation data from cBioPortal has been displayed on the BER module map using the pre-defined display mode as detailed in Table 1. The values represent average for all samples available in each dataset (A) BER in adenocortical carcinoma (B) Zoom in on initial steps of BER in adenocortical carcinoma, (C) Zoom in on execution step of BER in adenocortical carcinoma, (D) BER in ovarian serous cystadenocarcinoma, (E) Zoom in on initial steps of BER in ovarian serous cystadenocarcinoma, (F) Zoom in on execution step of BER in ovarian serous cystadenocarcinoma.

NaviCom can use aggregated values for all samples in each dataset from cBioPortal. This feature helps to generate interactive molecular portraits of cancers, facilitating comparison between cancer types. This is demonstrated in Figure 4, where the DNA repair map from ACSN collection is colored by expression and mutation data from four different types of cancers. The molecular portrait shows clear difference between the four types of cancer. Notable that Adenocortical Carcinoma (Figure 5B) data set is characterized by massive activation of cell cycle modules and high number of mutations, whereas the over three types of cancer are most probably less proliferative, as the cell cycle does not show up and over-activated (Figure 5A, C and D). Remarkably, the Acute Myeloid Leukemia dataset shows the least mutation frequencies (Figure 4A).

In addition, Figure 5 demonstrates that displaying the data on the sub-maps, in particular on the base excision repair (BER) module provides more subtle differences in regulation of this DNA repair pathway in different cancer types. Comparing BER pathway regulation in Adenocortical Carcinoma (Figure 5A) vs. Ovarian Serous Cystadenocarcinoma (Figure 5B) demonstrates that the pathway seems to be intact in the Adenocortical Carcinoma (Figure 5A), but not in Ovarian Serous Cystadenocarcinoma (Figure 5D) due to deregulation at the level of expression and multiple mutations in the key regulators of this pathway, especially the upstream initiators of this mechanism, NEIL1, 3, NTH1 and XPG (Figure 5B vs E). In addition, the executive step in BER process that is controlled by WRN and its co-factor PNK kinase is most probably inactive, because both WRN and PNK kinase are deregulated at the expression level and mutated in Ovarian Serous Cystadenocarcinoma (Figure 5F), but not in Adenocortical Carcinoma (Figure 5C). Collectively these observations can lead to a conclusion that Base Excision DNA Repair pathway is inactive in Ovarian Serous Cystadenocarcinoma.

The Supplementary Figure S3 shows more extensive visualization of the same data, using the whole ACSN map. Such comparisons allows to grasp the patterns in data across molecular mechanisms in a comprehensive manner, helping to highlight the specific deregulated processes in each type of cancer and highlight and deduce deregulated ‘hot area’ specific to each type of cancer. These signalling network-based molecular portraits and signatures that can be derived from them are of help for patients’ stratification. 

## Discussion

Combining together different types of high-throughput data provides a more complete description of alterations in a given condition, such as the set of genetic, epigenetic and post-translational alterations in cancer. Data visualization is a powerful method for quick grasping of the main points of interest in high-throughput data. These molecular descriptions placed in the context of biological processes depicted in a form of interactive maps, orient the researcher towards the deregulated mechanisms in studied samples and diseases. Signalling maps contain information about the connectivity between biological entities, therefore data visualization performed on top of network maps provides the possibility to retrieve entities relationship information to understand changes in molecular processes under different conditions.

Researchers in biology (and cancer biology) are often faced with the need to access public database resources for comparing their results with public molecular profiles (e.g. of tumor) and for analysing molecular observations in the context of signalling network map. Various omics data are available on public and local databases (4). However, there are no tools that support import of big datasets from these databases and displaying them on signalling network maps in efficient way and with optimized visualization settings. To answer to this demand, we developed NaviCom for automatic simultaneous display of multi-level data in the context of signalling network map, provided in a user-friendly manner.

We envisage several directions in further development of NaviCom. We plan to include network-based data processing methods such as geographical smoothing (over the neighbors in the network) to account for the extra information provided by the network.

NaviCom will soon provide pre-grouping of the data per various features available in cBioPortal, such as the grade of the disease, the age the patient, the survival status and generate directly a gallery of molecular portraits for various subtypes.

Similarly, NaviCom allows to selecting individual samples from a dataset or defining a group of samples. In order to ameliorate this feature, we will include into the NaviCom the possibility to filter tumoral samples by particular characteristics, for example presence of mutations, stage of disease, patient age, treatment type, time to relapse, etc. This filtering function will allow more selective, conclusive and narrowed analysis of the individual sample data.

One the demanded features is displaying type of mutation and amino acid substitution. This will be achieved by adding a label to the mutation glyph.

So far, NaviCom is optimized for one omics data resource to be linked to one type of signalling maps collection (cBioPortal to ACSN/NaviCell maps collections). However, NaviCom platform can be used to bridge any type of data and networks resources. In the near future we will extend the NaviCom platform to provide access to a wide range of omics databases (starting with ICGC, HGMB, CCLE etc.).

In addition, in order to allow a broader description of the molecular mechanisms implicated in the studied samples, signalling networks available in databases such as KEGG PATHWAYS (8), Reactome (7) SIGNOR (12) and others, will also be integrated and used for multi-level omics data analysis via NaviCom.

We will also address the current drawbacks of the tool, namely the significant time (several minutes) to create a molecular portrait, especially for large datasets and maps. This is connected with intrinsic delays required for data exchange in the current JavaScript implementation of the NaviCell API. We should notice that automated data fetching from cBioPortal and usage of predefined display settings to color the maps with the data makes the process of molecular portrait creation much faster compared to the time of manual manipulations, and does not require direct involvement of a user. However, for performing certain tasks, the response time of NaviCom might be still prohibitive. This can be ameliorated in the future by optimizing the way of internal data storage by NaviCom and applying smart caching methods.

The platform is already used for clinical samples comparison and will facilitate investigation of various biological conditions, tissue/cell types and diseases. For instance, NaviCom allows fast visualization of patients’ data in the context of maps and comparison with existing portraits from the associated gallery of molecular portraits of cancers different cancer types and subtypes available at the NaviCom website, helping for patient stratification.

## Supplementary Material

Supplementary Data
