# Supplementary material for: NaviCom: a web application to create interactive molecular network portraits using multi-level omics data
Source: Database (Oxford). 2017 Apr 2;2017:bax026. doi: 10.1093/database/bax026 (PMC5467574; doi:10.1093/database/bax026)

**Supplementary Figure 1**

**Molecular portrait of Glioblastoma visualized on Alzheimer’s signaling map**

Dataset: Glioblastoma TCGA Cell 2013

Sample: 3

Data types available: Copy Number, Expression, Mutations, Proteomics

Samples: 580

Visualization settings:

expression-map staining/ copy number-heat map/ mutations-blue triangle

**Triple display**


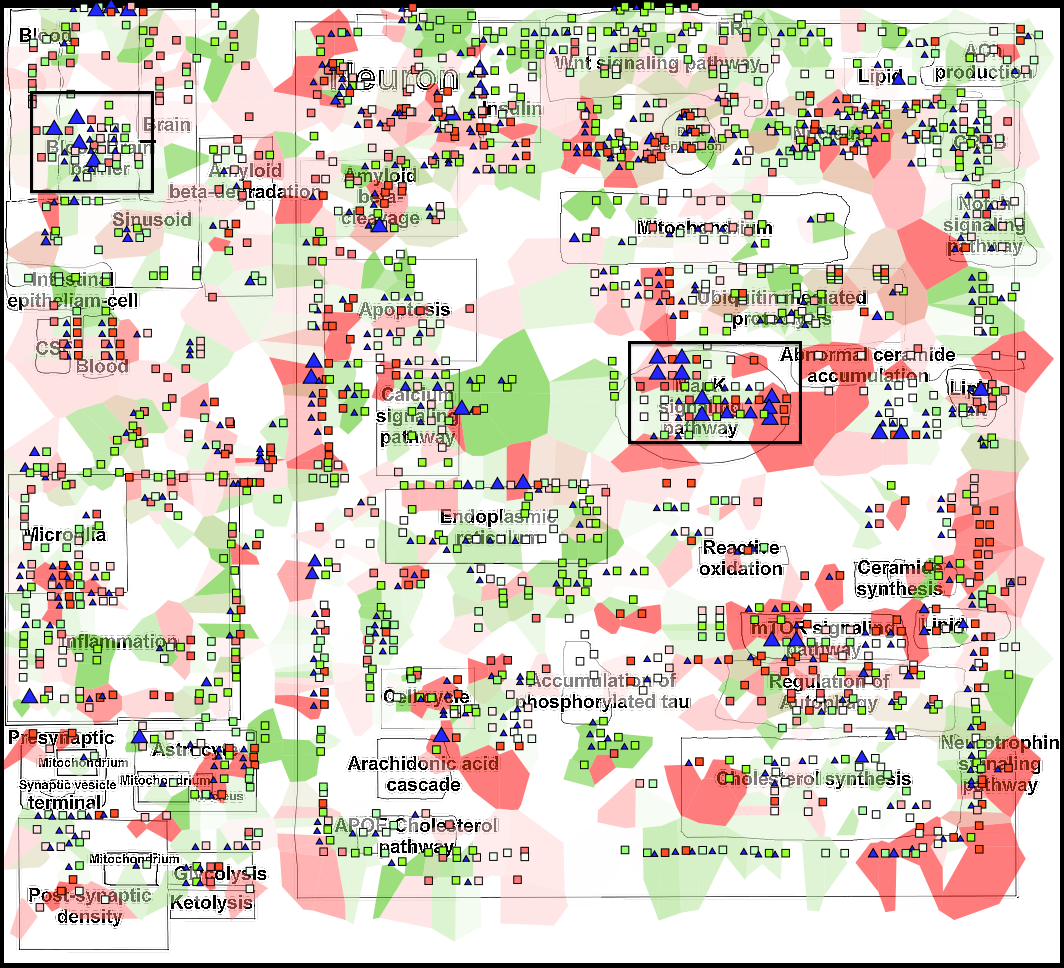


**MAPK pathway Blood-Brain barrier signaling**


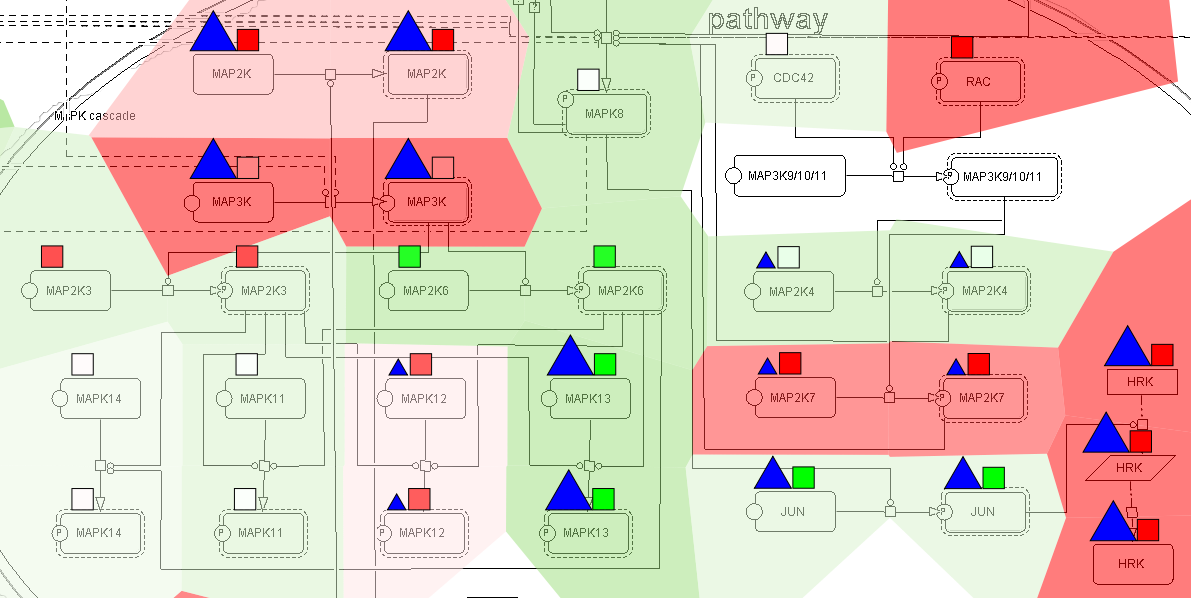

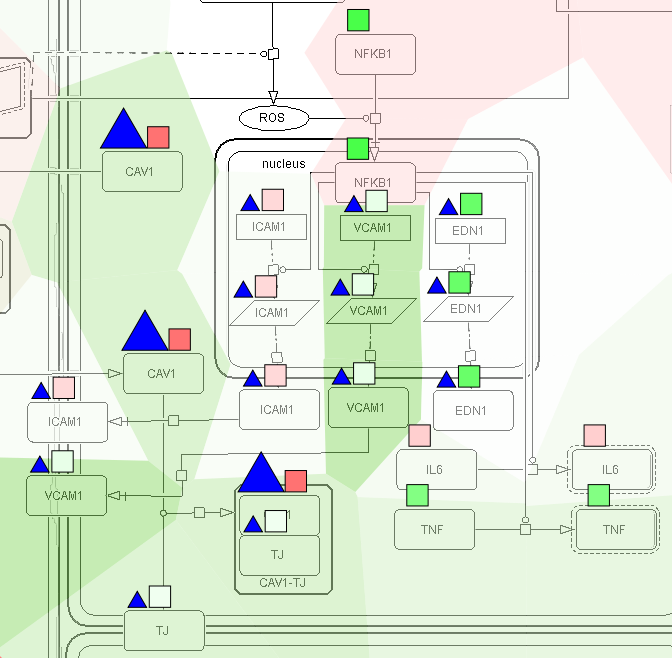


**Supplementary figure 2**

**Molecular portrait of Sarcoma visualized on Ewing’s sarcoma map**

Dataset: Sarcoma TCGA Provisional

Data types available: Copy Number, Expression, Methylation, Proteomics

Samples: 264

Visualization settings:

expression-map staining/ copy number-heat map/ methylation-pink diamond/ proteomics-yellow circle


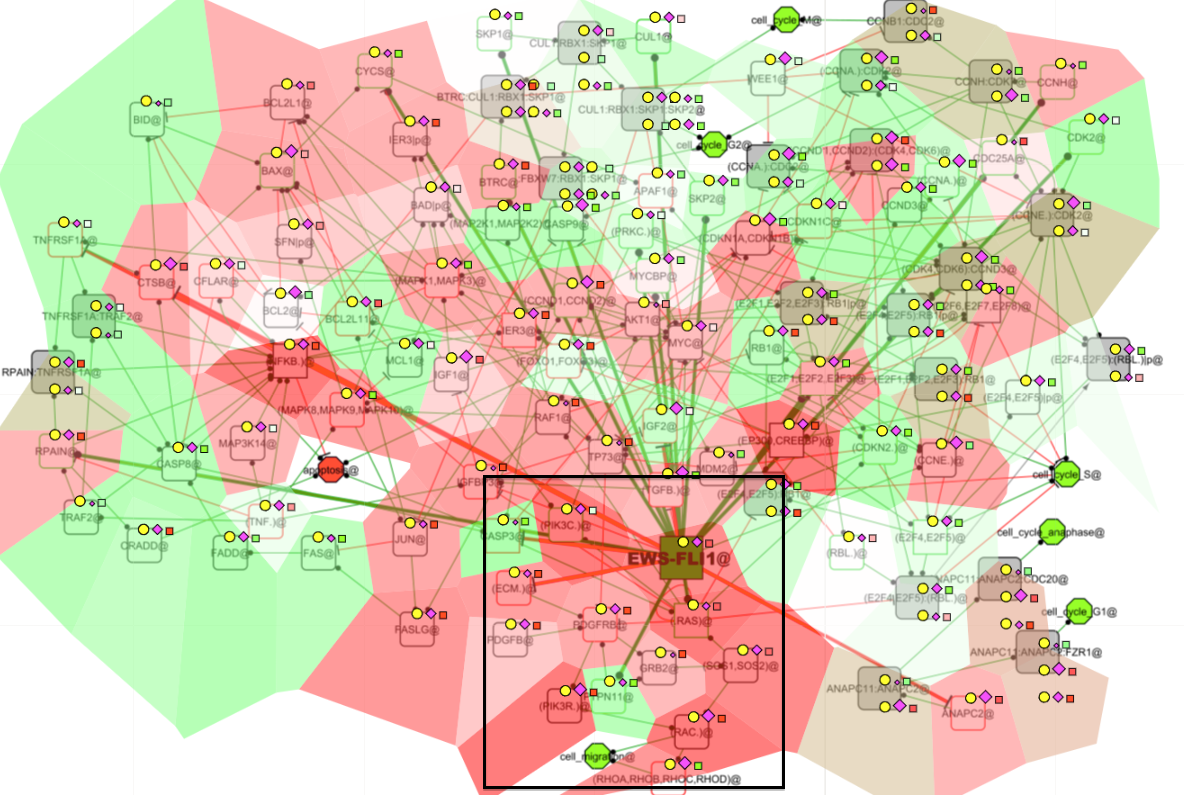


**Oncogenes directly regulated by EWS-FLI1**


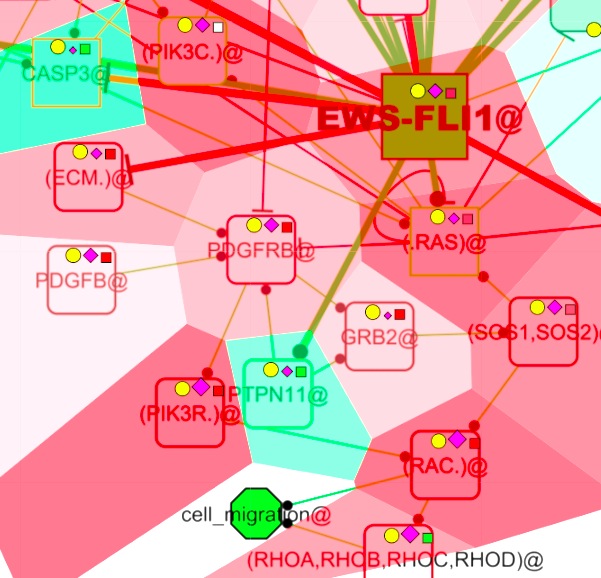


**Supplementary figure 3**

**Molecular portrait of several cancer types visualized on ACSN map**

Dataset: Acute Myeloid Leukemia TCGA NEJM 2013

Data types available: Copy Number, Expression, Mutations, Methylation, Mutations

Samples: 200

Visualization settings:

expression-map staining/ mutations-blue triangle


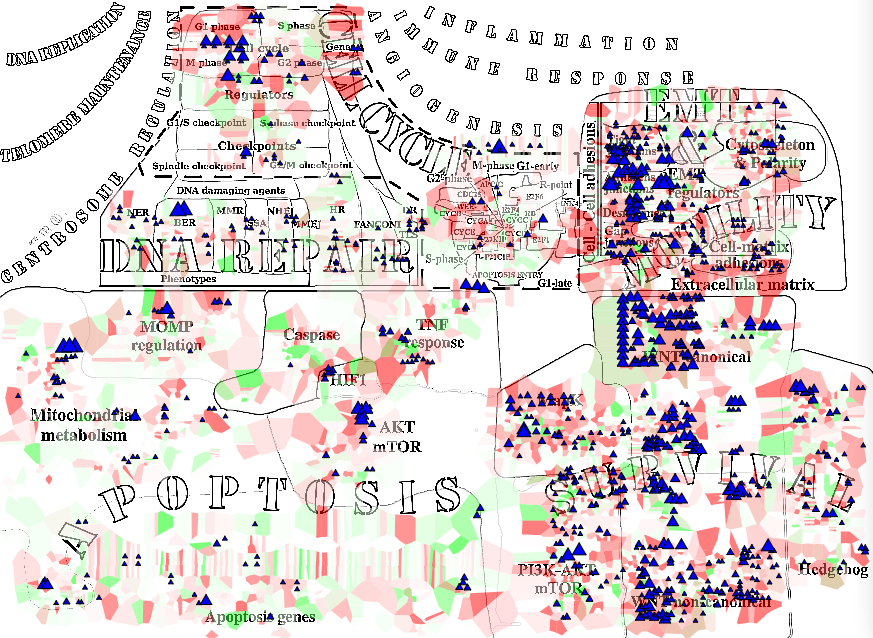


Dataset: Adenocortical Carcinoma TCGA Provisional

Data types available: Copy Number, Expression, Methylation, Mutations, Proteomics

Samples: 92

Visualization settings:

expression-map staining/ mutations-blue triangle


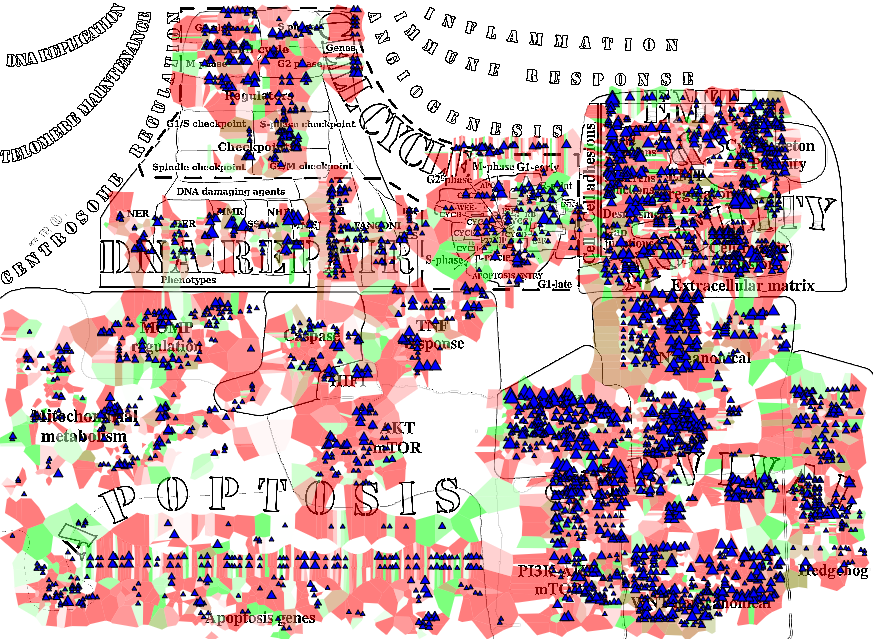


Dataset: Ovarian Serous Cystadenocarcinoma TCGA Nature 2011

Data types available: Copy Number, Expression, Methylation, Mutations

Samples: 557

Visualization settings:

expression-map staining/ mutations-blue triangle


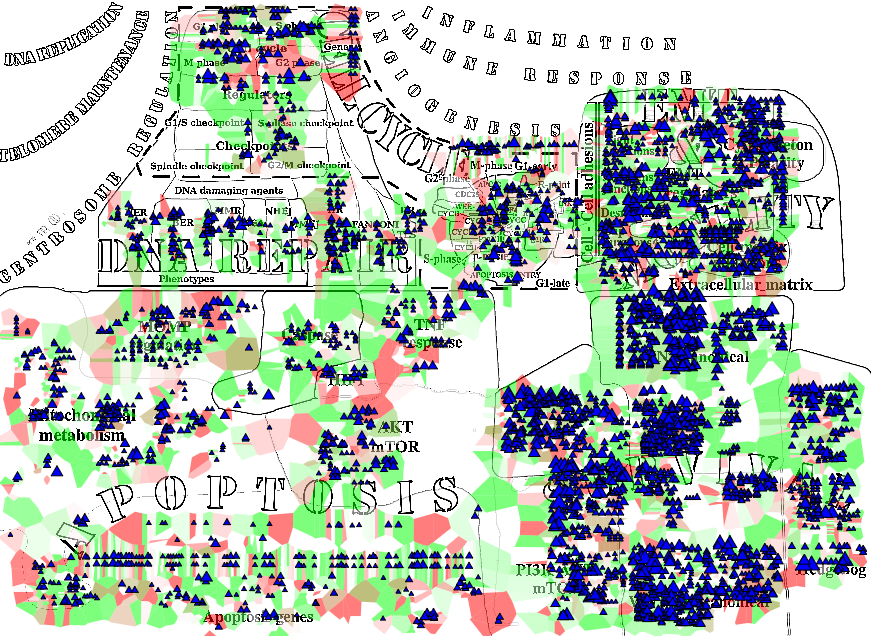


Dataset: Glioblastoma TCGA Cell 2013

Data types available: Copy Number, Expression, Mutations, Proteomics

Samples: 580

Visualization settings:

expression-map staining/ mutations-blue triangle


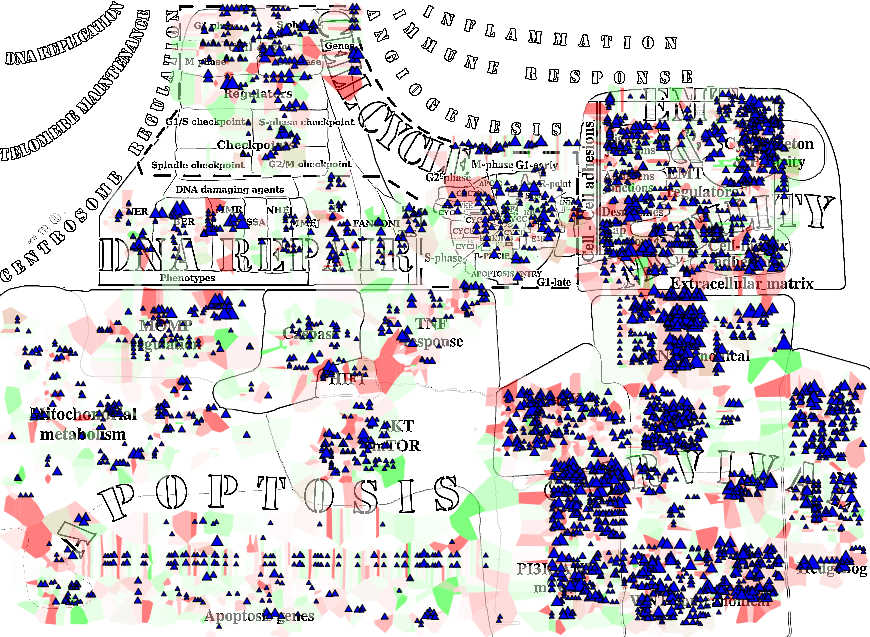

Supplement: Supplementary Data [file bax026_Supp.zip › Dorel_etal_SupplementaryFigures_DATABASE_BiocurationVirtualIssue_REVISED.docx]
