# Supplementary material for: NaviCom: a web application to create interactive molecular network portraits using multi-level omics data
Source: Database (Oxford). 2017 Apr 2;2017:bax026. doi: 10.1093/database/bax026 (PMC5467574; doi:10.1093/database/bax026)
Supplement: Supplementary Data [file bax026_Supp.zip › Dorel_etal_SupplelmentaryTable_DATABASE_BiocurationVirtualIssue.docx]

**Supplementary table 1**

**Using NaviCom from the command line**

| Download and save data from cBioportal using cBioFetchR | # Load the cBioFetchR package and connect to cBioPortal  library ( cBioFetchR)  conn = cBioConnect("http://www.cbioportal.org/")  # Select the study id  listStudies(conn, " ovarian")  # Get the data from cBioportal and prepare the data for  NaviCell export  datas = cBioNCviz("ov_tcga_pub", genes_list="http://acsn.curie.fr/files/acsn_v1.1.gmt", name="",  method = "profiles")  # Export in a format readable by the Python module  saveData(datas) |
| --- | --- |
| Import data in python | from navicom import *  # Prepare the connex ion  nc = NaviCom(map_url='https://navicell.curie.fr/  navicell/maps/cellcycle/master/index.php',  browser_command="google-chrome %s",  display_config = DisplayConfig(5, na_color="ffffff"))  # Load data for the visualisation  nc.loadData(fname="Ovarian_tcga_pub") |
| Select and display data on the NaviCell map | nc.listData() # Get the data a v a i l a b l e  nc.listAnnotations() # Get the groups a v a i l a b l e  # Display the data on the map  nc.completeDi splay()  # Display for a specific group  nc.completeDisplay(sample="GRADE: DiseaseFr ee") |

The activity of NaviCom requires the R package cBioFetchR and the Python module navicom. With those packages, it is possible use NaviCom with the command line, granting more flexibility in various ways, such as a more precise configuration of the display in NaviCell or the list of genes downloaded from cBioPortal.
